# Comparison of digital and silicone impressions for single-tooth implants and two- and three-unit implants for a free-end edentulous saddle

**DOI:** 10.1186/s12903-021-01836-1

**Published:** 2021-09-23

**Authors:** Koudai Nagata, Kei Fuchigami, Yurie Okuhama, Kana Wakamori, Hayato Tsuruoka, Toshifumi Nakashizu, Noriyuki Hoshi, Mihoko Atsumi, Katsuhiko Kimoto, Hiromasa Kawana

**Affiliations:** 1 Department of Oral and Maxillofacial Implantology, Kanagawa Dental University, 82 Inaoka-cho, Yokosuka, 238-8580 Japan; 2 Department of Dental Laboratory, Kanagawa Dental University Hospital, Yokosuka, Japan; 3 Division of Prosthodontics and Oral Implantology, Department of Oral Interdisciplinary Medicine, Graduate School of Dentistry, Kanagawa Dental University, Yokosuka, Japan

**Keywords:** Dental implant, Digital impression, Digital workflow, Implant impression, Intraoral scanner

## Abstract

**Background:**

The use of intraoral scanners (IOS) has facilitated the use of digital workflows for the fabrication of implant-supported prostheses not only for single missing teeth, but also for multiple missing teeth. However, the clinical application of IOS and computer-aided design/manufacturing (CAD/CAM) in implant-supported prosthodontics remains unclear. This study aimed to compare the accuracy of digital and silicone impressions for single-tooth implants for bounded edentulous spaces and two-unit and three-unit implant-supported fixed dental prostheses for free-end edentulous spaces.

**Methods:**

This study enrolled 30 patients (n = 10 for each of the three groups) with an average age of 61.9 years. Conventional silicone-based and digital IOS-based impressions were made for all patients, and the implant superstructures were fabricated. We measured the scan-body misfit and compared the accuracy of the impressions for single-unit, two-unit, and three-unit implant prostheses with a bounded edentulous space by superimposing the standard triangulated language (STL) data obtained from IOS over the STL data of the plaster model used for final prosthesis fabrication. The scan bodies of the superimposed single-molar implant, two-unit implant prosthesis without teeth on the mesial side, two-unit implant prosthesis without teeth on the distal side, three-unit implant prosthesis without teeth on the mesial side, and three-unit implant prosthesis without teeth on the distal side were designated as A, B1, B2, C1, and C2, respectively. The misfit for each scan body was calculated and the accuracies were compared using the Tukey–Kramer method.

**Results:**

The average scan-body misfit for conditions A, B1, B2, C1, and C2 was 40.5 ± 18.9, 45.4 ± 13.4, 56.5 ± 9.6, 50.7 ± 14.9, and 80.3 ± 12.4 μm, respectively. Significant differences were observed between the accuracies of A and B2, A and C2, and C1 and C2 (P < 0.001).

**Conclusions:**

IOS and CAD/CAM can find clinical applications for implant-supported prostheses of up to three units for a bounded edentulous saddle. The use of IOS could render implant treatment easier, benefiting both the surgeons and patients. Prosthesis maladjustment may lead to peri-implantitis and prosthetic fracture. Therefore, further validation of the accuracy of IOS impressions is required in patients with multiple missing teeth in long-span implant prostheses.

## Background

The use of digital technology in clinical dentistry has become widespread in recent years, enabling dentists to provide better care to their patients. The use of intraoral scanners (IOS) and computer-aided design/manufacturing (CAD/CAM) systems for the fabrication of crowns and bridges has facilitated the digitization of almost all the processes involved in prosthodontic treatment [1–3].

The use of IOS has facilitated the use of digital workflows for the fabrication of implant-supported prostheses not only for single missing teeth, but also for multiple missing teeth [4, 5]. The benefits of using IOS include reduced patient discomfort, shorter chair time, shorter prosthesis fabrication time, and the ability to store patients’ biometric information as data [6, 7]. However, only a few clinical studies have reported on the utility of IOS for implant-supported prosthetic treatment. Most previous studies conducted in vitro comparisons of precision and accuracy between the digitally fabricated and conventional models used for implant-supported prosthetic treatment [8–10]. Kocaağaoğlu et al. [11] compared the marginal misfit of three-unit frames made with conventional and digital impressions and found that the mean marginal misfit of conventional impressions was 98.8 ± 16.43 µm, whereas that of the digital impression was 65.14 ± 18.05 µm. The authors reported that the misfit was larger with the conventional method. The extent to which IOS and CAD/CAM can be used clinically for the fabrication of implant-supported prostheses remains unclear.

In clinical practice, it is impossible to ascertain the impression method with the highest accuracy when comparing the traditional silicone method with the digital method. Thus, evaluating the misfits of the prostheses fabricated with each method affords the only opportunity to verify their respective accuracies. The purpose of this study was to compare the clinical accuracy of digital and silicone impressions made for single-tooth implants with teeth present on both sides and two- and three-unit implant-supported fixed dental prostheses with teeth present only on one side of the edentulous space.

## Methods

### Study design

**Fig. 1:**
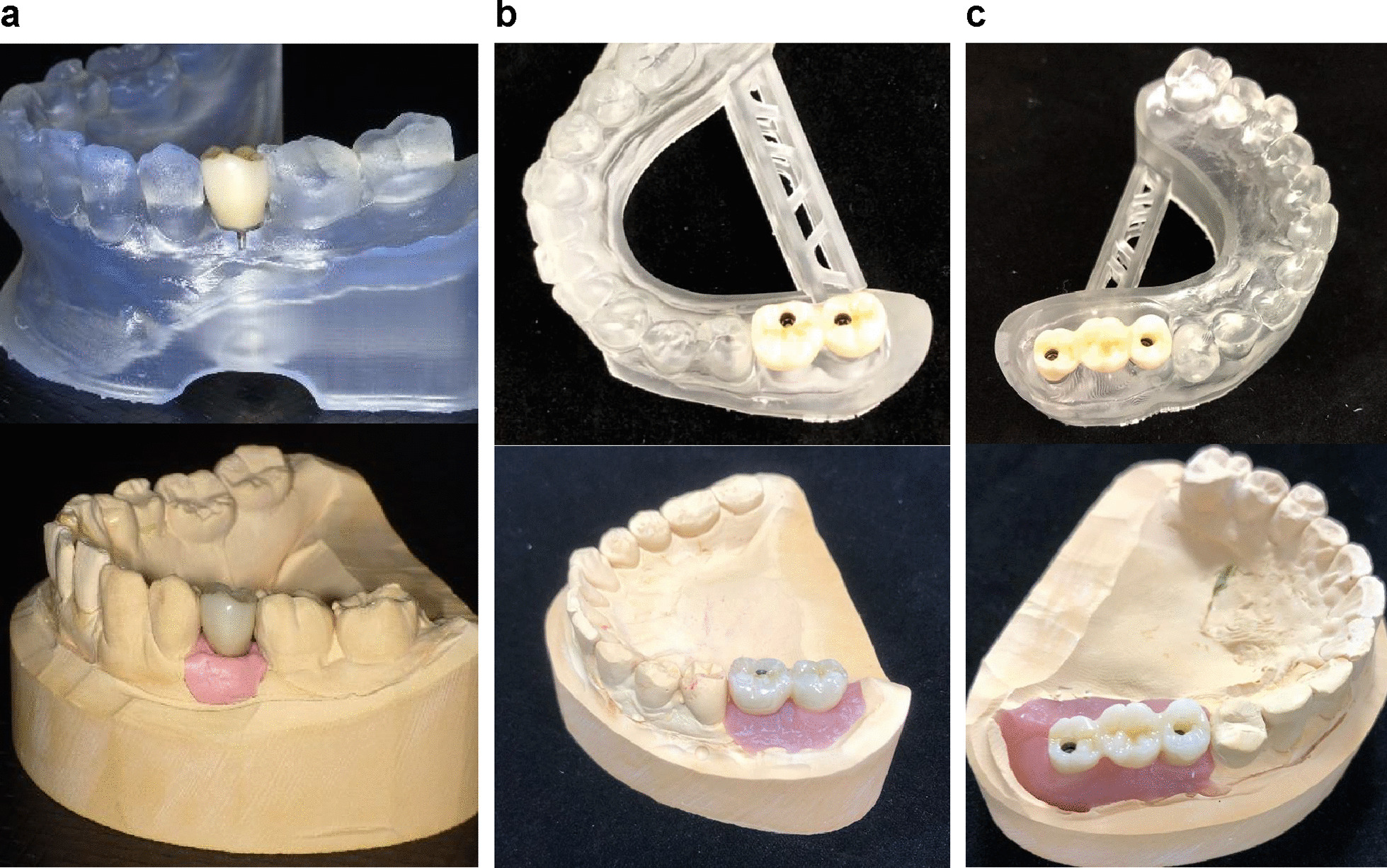
The superstructures were fabricated from impressions obtained using an intraoral scanner (IOS) and silicone material. **a** Single-tooth implants. **b** Two-unit implants. **c** Three-unit implants

This study was approved by the ethics committee of Kanagawa Dental University (approval number 555). Research involving human participants, were performed in accordance with the Declaration of Helsinki and with the relevant guidelines and regulations. Written informed consent was obtained from all participants. Digital IOS-based impressions and conventional silicone-based impressions were made for all patients, and the implant superstructures were fabricated (Fig. 1). The accuracy of the impressions made for single-tooth implants with teeth present on both sides and two-unit or three-unit implant-supported fixed dental prostheses with teeth present only on one side of the edentulous space was compared by superimposing the standard triangulated language (STL) data obtained from the IOS and the STL data from plaster models retrieved from the silicone impressions.

### Participants

Thirty patients, with an average age of 61.9 years, who agreed to undergo implant treatment at our university hospital, were included according to the following three criteria: missing single molar with natural teeth on both sides (n = 10), two missing teeth with natural teeth present on only one side of the edentulous space (n = 10), and three missing teeth with natural teeth present on only one side of the edentulous space (n = 10). All patients were treated by the same dentist from the Department of Oral and Maxillofacial Implantology, and all laboratory procedures were performed by the same dental technician.

Patients were included in the study only if they did not have a history of systemic diseases, were non-smokers, and did not require bone grafting.

### Surgical procedure

All implant placements were performed via freehand insertion and in accordance with the implant system protocol [12]. The Straumann® φ 4.1 mm (standard plus implant, bone level tapered implant, Basel, Switzerland) implant system was used in this study. All implant surgeries were performed using the two-stage method, and the post-surgical recovery period was 2 months.

### Evaluation and measurement

The open tray (made of resin) technique with silicone-based impression material was used as a conventional method to acquire STL data. An impression coping was placed over the implant in the oral cavity, and precise impressions were made using a silicone-based material (Aquasil Ultra®; Dentsply Sirona, York, PA). Subsequently, a scan body (Mono Scanbody RC, RN, Straumann®, Basel, Switzerland) was mounted over the plaster model (New Fujirock®; GC, Tokyo, Japan), followed by scanning with a 3D scanner (Ceramill Map400®; Amann Girrbach, Vienna, Austria), and conversion to STL data. For STL data acquisition by digital impression technique, a scan body was placed in the patient's mouth and digital impressions were taken using an IOS (Trios 3®, 3Shape; Copenhagen, Denmark). Using Geomagic Control® (3D Systems, Washington, DC), the STL data obtained from the conventional method and the STL data obtained from the digital impression method were superimposed to measure the scan body misfit. The superposition of the two STL data sets was performed after trimming the excess data, followed by manual alignment based on three landmarks, and best-fit registration was used for greater accuracy. Three points were randomly selected from the data of the superimposed scan body, and the average value was considered as the misfit between the digital and conventional impressions. The positions of the scan body mounted on the plaster model and scan body mounted in the oral cavity were set to be equivalent.

**Fig. 2:**
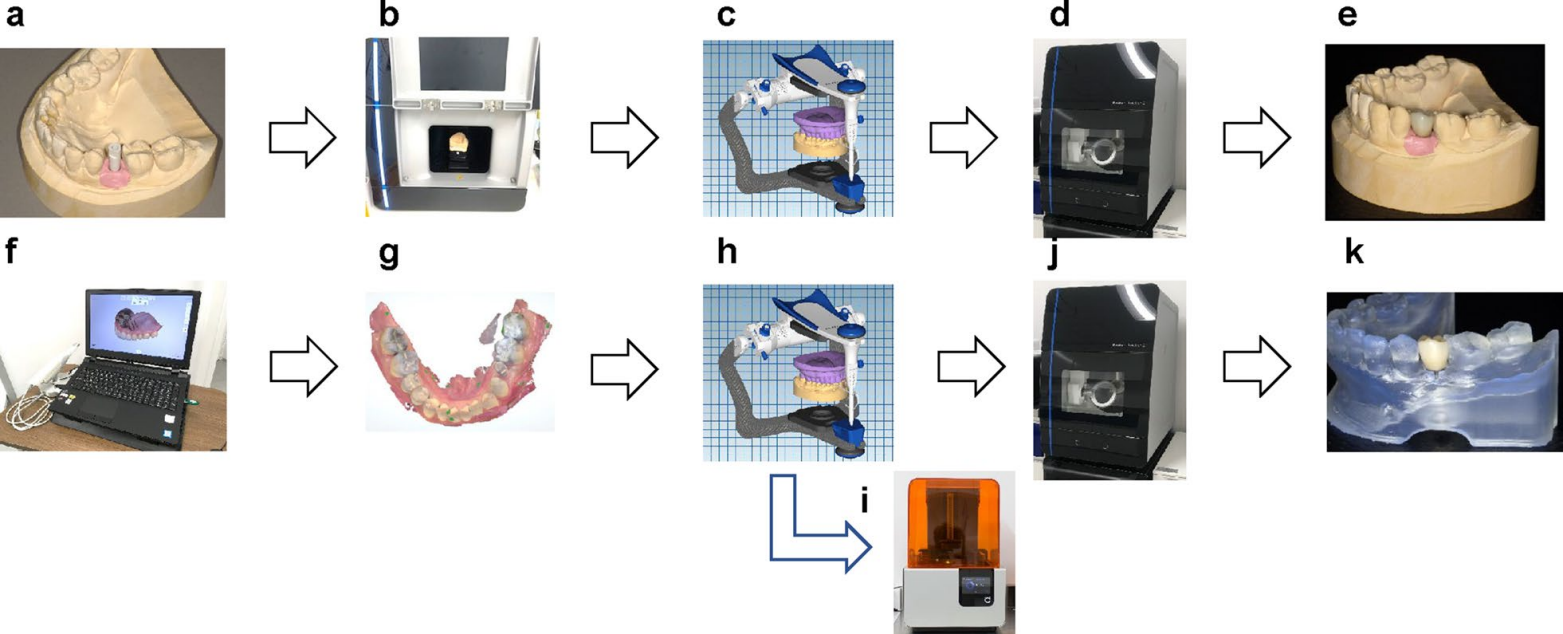
Workflow of the conventional and digital impression methods. **a** Impression making procedure with silicone and making implant models. **b** Scanning a model with the 3D scanner. **c** Designing the superstructure using CAD. **d** Cutting out the superstructure using CAM. **e** Completion of the superstructure. **f** Digital impression with Trios. **g** Obtaining intraoral data from the digital impressions. **h** Designing the superstructure using CAD. **i** Fabrication of the resin models using a 3D printer. **j** Milling the superstructure using CAM. **k** Completion of the superstructure. *CAD* computer-aided design, *CAM* computer-aided manufacturing, *3D* 3-dimensional

CAD software (Exocad®; Exocad, Berlin, Germany) and CAM (Ceramill motion2®; Amann Girrbach, Wien, Austria) were used to design and fabricate the superstructure, respectively (Fig. 2).

A fixed screw-retained implant superstructure composed of zirconia (Ceramill Zolid®, Amann Girrbach, Vienna, Austria) was used. All silicone and IOS impressions were made at the implant level. The abutment was not interposed with the superstructure in single implants, while the abutments were interposed with the superstructure in implants with two or three units. All superstructures were fabricated with zirconia.

**Fig. 3:**
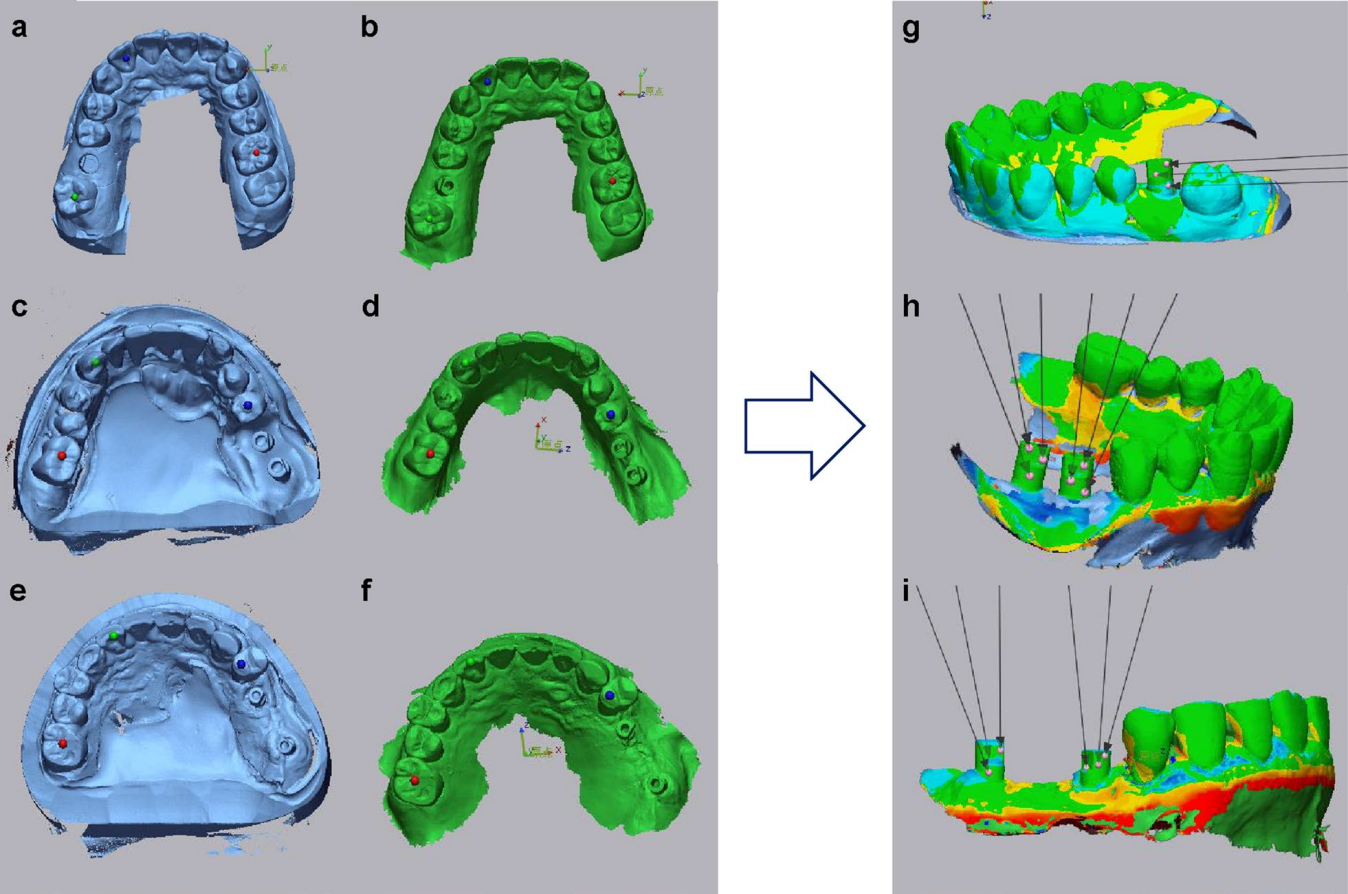
Measurement of the misfit of the scan body using Geomagic Control. **a** STL data of a plaster model of a single-tooth implant. **b** STL data of a single-tooth implant obtained from the IOS. **c** STL data of a plaster model of a two-unit implant. **d** STL data of a two-unit implant obtained from the IOS. **e** STL data of a plaster model of a three-unit implant. **f** STL data of a three-unit implant obtained from the IOS. **g** The scan-body misfit was designated as A. **h** The misfit on the mesial side was designated as B1 and that on the distal side as B2. **i** The misfit on the mesial side was designated as C1 and that on the distal side as C2. *STL* standard triangulated language, *IOS* intraoral scanner

The superimposed images of the scan bodies in patients with single-tooth molar implants bound by teeth on the mesial and distal sides, were designated as A. The superimposed images of the scan bodies in patients with two-unit implant prosthesis with no teeth on the mesial side were designated as B1, and those without teeth on the distal side were designated as B2. The superimposed images of the scan bodies in patients with three-unit implant prosthesis without teeth on the mesial side were designated as C1 and those without teeth on the distal side were designated as C2. The misfit for each scan body was calculated (Fig. 3) and the accuracies were subsequently compared.

The implantation sites of two-unit prostheses included the second premolar and first molar, or the first molar and second molar, while the sites of three-unit prostheses were the first premolar, first molar/second premolar, and the second molar. The average distance between the centers of the implants was 9.9 mm in the two-unit prostheses and 15.3 mm in the three-unit prostheses.

### Statistical analysis

G-Power (version 3.1.9.2) was used to perform the one-way analysis of variance. We calculated the sample size required to obtain 80% of the effect size of 0.4 at α = 0.05. A total sample size of 60 people was needed. However, since there were not many patients who met the requirements, the total sample size was set at 30 in this study.

We considered a P-value of < 0.05 to indicate significance. BellCurve for Excel (Social Survey Research Information Co., Ltd., Tokyo, Japan) was used to perform statistical processing using the Tukey–Kramer method.

## Results

### Misfits in scan bodies

The average misfit was calculated in all the conditions. The average misfit in conditions A, B1, B2, C1, and C2 was 40.5 ± 18.9, 45.4 ± 13.4, 56.5 ± 9.6, 50.7 ± 14.9, and 80.3 ± 12.4 μm, respectively.

### Comparison of scan-body misfits between the different conditions

**Fig. 4:**
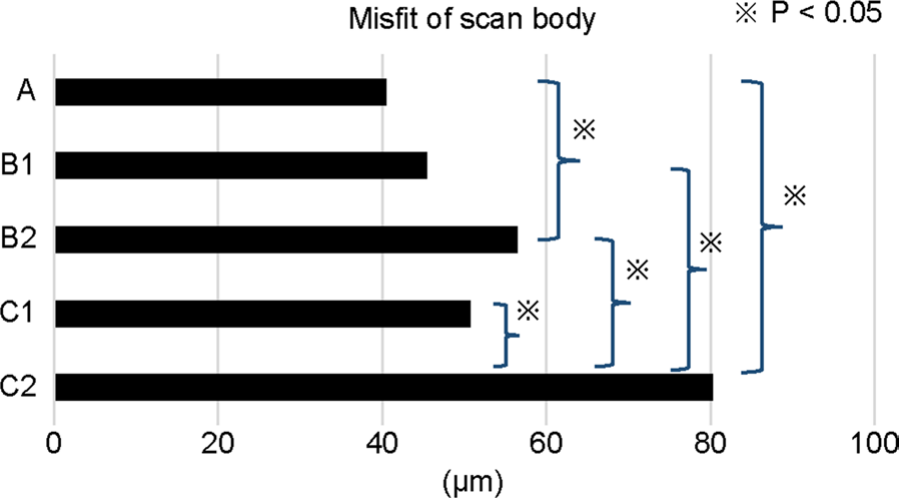
Comparison of each scan-body misfit. The scan bodies of the superimposed single-molar implant, two-unit implant prosthesis without teeth on the mesial side, two-unit implant prosthesis without teeth on the distal side, three-unit implant prosthesis without teeth on the mesial side, and three-unit implant prosthesis without teeth on the distal side were designated as A, B1, B2, C1, and C2, respectively

The misfits in the different conditions were analyzed as a measure of accuracy for the comparison among the different conditions. A significant difference was observed between A and B2, and between A and C2 (P < 0.001). The degree of misfit was the highest in C2 when compared to all the other conditions (Fig. 4).

## Discussion

This study compared the accuracy of digital impressions made using IOS with that of conventional impressions made using silicone impression materials. The results showed that the scan-body misfit between the two methods was the highest for C2, while condition A had the smallest misfit.

The current implant-supported prosthodontic treatment protocol using silicone impressions entails the placement of the impression coping in the oral cavity, followed by the acquisition of the silicone-based impression. Subsequently, a plaster model is retrieved from the impression and the scan body is placed on the plaster model, which is then read by a 3D scanner, followed by the CAD/CAM process. Although the fabrication of zirconia superstructures is predominantly performed using this method, there have been concerns that the deformation of the silicone impression material and expansion of the plaster model could affect the accuracy of the prosthesis [13–15].

However, digital impressions using IOS have facilitated the designing of zirconia superstructures using CAD and machining using CAM [16, 17], which have streamlined the process of prosthesis fabrication; thus, reducing the treatment time, laboratory time, and errors [8, 18]. Several studies have reported on the performance of IOSs. Seelbach et al. [1] compared the digital and conventional impression techniques for crowns and bridges and found no difference among the fit of crown margins fabricated using the three types of IOSs (Lava Chairside Oral Scanner, Chairside Economical Restoration of Esthetic Ceramic, and iTero) and the conventional method. Systematic reviews conducted by Chochlidakis et al. [19] and Ahlholm et al. [20] have also found that the fit of prostheses such as crowns and bridges fabricated from conventional or digital impressions was good, and no difference was observed between them. Flügge et al. [21] conducted a systematic review of 79 studies on implant impression making and found that only 20 studies focused on digital impressions, while most studies used plaster or other models. Fukazawa et al. [22] placed a ball attachment on study models and measured the respective accuracies of the IOS and laboratory scanner in a study that investigated the accuracy of IOSs in implant treatment. The misfit of the IOS was greater in the model with a distance of 18.4 mm between the centers of the ball attachments than that in the model with a distance of 9.6 mm between the centers of ball attachments. Flügge et al. [23] also evaluated the IOS using study models and reported that the accuracy of the IOS decreased with the increase in the distance and angle between the scan bodies. Tan et al. [24] also reported that a short interdental distance between implants was related to higher accuracy using a maxillary edentulous model. All of the above-mentioned studies were based on models and reported a decrease in accuracy as the distance between the implant bodies increased.

Our results showed that there were no significant differences between A and B1 or A and C1, whereas significant differences were observed between A and B2 and between A and C2. Condition A, where the mesial and distal teeth adjacent to the (edentulous) defect were present, showed the smallest misfit, followed by B1 and C1, which were located close to the remaining teeth. Conversely, the misfit was larger in B2 and C2, where the distance from the landmark was greater. The results were similar to those reported by previous studies. Few clinical studies have reported on the accuracy of digital impressions in implant treatment. Delize et al. [25] compared superstructures fabricated using digital impressions and the conventional method based on the occlusal and interdental contact in 31 patients with a single missing tooth. They reported that the respective fits of the superstructures fabricated by both processes were good and lacked a significant difference. Mühlemann et al. [26] measured the accuracy of impressions in five patients with a single missing tooth with teeth on both sides (of the edentulous space) using the conventional method and three types of IOSs (iTero Cadent, Lava True Definition, and Trios 3Shape). They reported that the scan-body misfit was 32.7 ± 11.6 μm using the conventional method, and 57.2 ± 32.6 and 88.6 ± 46 μm using the iTero and Trios systems, respectively. Gedrimiene et al. [27] compared conventional silicone-based and IOS-based digital impressions in six patients with multiple missing teeth with teeth on both sides of the edentulous space and found that the misfit of the scan body was 70.8 ± 59 μm and that IOSs can be used in implant-supported prostheses with up to four units. Studies investigating the prosthetic fit have reported that cement spaces lower than 100 μm were acceptable [28–30]. Al-Meraikhi et al. [31] investigated the fit of the abutment on the implant body and reported that spaces lower than 135 μm were acceptable.

In the present study, the average misfit for three-unit prostheses with no remaining teeth on the distal side was found to be 80.3 ± 12.4 μm. We suggest that digital impressions using IOS could be indicated in clinical practice for implant prostheses of up to three units, based on the findings of the current and previous studies. The greater the angular error of the implant body, the lower the accuracy of the IOS impression; therefore, guided implant surgery is among the methods that can ensure accurate prosthetic treatment. The fit of the superstructure in implant prostheses is likely to vary in clinical practice depending on various factors, such as the condition of the edentulous space, the number of units in the prosthesis, and whether powder was used when taking impressions with IOS.

Maladjustment of the prosthesis may lead to peri-implantitis and prosthetic fracture. Therefore, further validation of the accuracy of IOS impressions is required in patients with multiple missing teeth.

A limitation of this study is the small sample size for each condition; the eligibility criteria made it difficult to include more patients in each group. Another limitation is that the bone resorption, loosening of the superstructure, and failure of the superstructure for each impression type could not be evaluated using follow-up examinations.

Finally, a single implantologist was in charge of the entire process from implant placement to prosthetic treatment. After placement of the superstructure, maintenance was performed every three or six months. For patient self-care, he recommended thorough brushing and chlorhexidine mouthwash. Owing to the current world situation, the medical community is baffled by the coronavirus disease 2019 (COVID-19) pandemic. Severe acute respiratory syndrome coronavirus 2 adheres to the oral cavity and respiratory tract, and dental treatment may increase the risk of infection; therefore, knowledge regarding its prevention is necessary [32, 33]. It is well known that chlorhexidine is effective in the treatment of gingivitis and the prevention of peri-implantitis [34]. There are also reports that chlorhexidine exerts a preventive effect on COVID-19 [35]. It may be possible to reduce the risk of infection for both the dentist and patient by having patients wash their mouths with chlorhexidine before treatment. In addition, thorough infection prevention in medical care systems can be a measure against not only COVID-19 but also various other infectious diseases [36]. Whether digital technologies, such as IOS, can reduce the risk of infection compared to conventional methods will be a subject of future research.

## Conclusions

The application of IOS for implant treatment could render treatment easier, which is beneficial for both surgeons and patients. However, its application in implant-supported prostheses has been investigated by only a few clinical studies; thus, the extent to which digital technologies, such as IOS, can be applied in routine clinical practice is unclear. Our study suggested that digital impressions made using IOS can be used for the fabrication of implant prostheses of up to three units with teeth present on only one side of the edentulous space. We will endeavor to verify the clinical results in long-span implant prostheses in the future.

## Data Availability

The datasets obtained and analyzed during the current study are available from the corresponding author upon reasonable request.

## Abbreviations

3D: Three-dimensional
CAD/CAM: Computer-aided design/manufacturing
IOS: Intraoral scanners
STL: Standard triangulated language
